# LFP-LOC: an LFP power–based method for validating the anatomical placement of high-density neural probes in rodents

**DOI:** 10.3389/fnins.2026.1816533

**Published:** 2026-05-11

**Authors:** Alberto Perna, Raffaele Adamo, Matteo Vincenzi, Gian Nicola Angotzi, João Filipe Ribeiro, Luca Berdondini

**Affiliations:** 1 Microtechnology for Neuroelectronics Laboratory, Fondazione Istituto Italiano di Tecnologia, Genova, Italy; 2 The Open University Affiliated Research Centre at Istituto Italiano di Tecnologia (ARC@IIT), Genova, Italy

**Keywords:** anatomical localization, brain atlas, CMOS neural interfaces, local field potentials, online probe placement, power spectral density, SiNAPS neural probes, spatial distribution

## Abstract

High-density CMOS-based neural probes provide unprecedented spatiotemporal resolution for in-vivo electrophysiology, yet accurate validation of implant position remains challenging. Here we present LFP-LOC, a simple and interpretable method for intraoperative validation and refinement of probe anatomical location based on the spatial distribution of local field potential (LFP) power. Using spontaneous activity recordings performed in rodents, we compute power spectral densities in canonical LFP bands and apply dimensionality reduction and clustering to identify electrodes with shared spectral signatures. Across multiple implant sites, probe technologies, electrode layouts, and experimental conditions, the resulting clusters consistently align with anatomical boundaries. Applied to high-density probes with up to 1,024 electrodes/channels and sub-30 μm pitch, power features converge within approximately 20 s of recordings, allowing online intraoperative assessment. By leveraging the robust relationship between LFP power and brain structure, LFP-LOC enables rapid validation and adjustment of probe placement during surgery, complements histological validation, and may facilitate mesoscale electrophysiological studies.

## 1 Introduction

Over the past decade, the advent of high-density CMOS-based neural probes has drastically increased both the spatial resolution and the field of view available for the *in-vivo* investigation of neural dynamics (Jun et al., 2017; Raducanu et al., 2017; Steinmetz et al., 2021; Lee et al., 2022; Perna et al., 2023; Angotzi et al., 2025; Ribeiro et al., 2025). However, acquiring precise knowledge of implant location during electrophysiological recordings remains challenging. In most experiments, probe placement is planned pre-operatively and validated *post hoc* using *ex-vivo* histology, which is labor-intensive, error-prone, and highly dependent on operator expertise (Shamash et al., 2018). Moreover, histological reconstruction typically reveals only the macroscopic trajectory of the probe shank, often enhanced using fluorescent dyes (Dicarlo et al., 1996; Guo et al., 2017; Shamash et al., 2018; Liu et al., 2021) or electrolytic lesions (Dicarlo et al., 1996; Sofroniew et al., 2015), rather than the precise location of individual recording sites relative to anatomical structures. Probe trajectories are rarely contained within an individual tissue slice, and sections are typically compared to fixed-angle reference images (coronal or sagittal) neglecting likely misalignments in cut angle (Shamash et al., 2018; Lauridsen et al., 2022). The cutting and processing steps can also introduce tissue deformation (Borg et al., 2015; Liu et al., 2021), further compromising spatial accuracy. As a result, reliably assigning individual electrodes to specific anatomical regions through immunohistology is challenging. In addition to these limitations, histological validation is not always feasible, depending on the animal model or in clinical applications.

Alternative approaches aimed at visualizing probe placement within the intact brain include ex-vivo tissue clearing combined with light-sheet microscopy (Jensen and Berg, 2016; Ueda et al., 2020; Liu et al., 2021; Catanese et al., 2024) and *in-vivo* imaging based on the combination of computed tomography (CT) and magnetic resonance imaging (MRI) scans (Nahm et al., 1994; Rangarajan et al., 2016; Király et al., 2020; Censoni et al., 2022). While tissue clearing enables whole-brain visualization at high spatial resolution, it is inherently time-consuming, as the clearing process can require several days, and is restricted to post-hoc analyses. Conversely, CT–MRI–based localization demands specialized and costly instrumentation that is not always available in neuroscience labs and high-density CMOS probes have not yet been optimized for MRI compatibility.

A different and complementary strategy for validating implant location is to leverage the rich neural signals provided by high-density probes to establish correlations with cytoarchitectural and anatomical features. Electrophysiological activity in the action potential (AP) band has been used to identify putative neuronal cell types (Shreejoy et al., 2015; Buccino et al., 2018; Gouwens et al., 2019; Lee et al., 2021; Petersen et al., 2021) and to infer the brain regions to which they belong (Yu et al., 2024). However, this approach is highly region dependent and relies on spike detection and sorting algorithms, which are computationally demanding and prone to errors (Rey et al., 2015; Buccino et al., 2020). In contrast, local field potentials (LFPs) may constitute a more robust solution for implant localization. Notably, LFPs allow to estimate single-unit firing rates (Jackson and Hall, 2017) and to develop high-performance, chronically stable brain-machine interfaces (Rickert et al., 2005; Waldert et al., 2009; Flint et al., 2012; Stavisky et al., 2015), underscoring their rich information content.

Recently, a self-supervised learning framework, *Lfp2vec* (He et al., 2025), was introduced to infer anatomical regions directly from LFP recordings. This method clusters electrodes by extracting intrinsic structure from LFP data using contrastive learning. However, this approach lacks interpretability, requires a training phase, and depends on a supervised fine-tuning stage to assign clusters to anatomical regions, affecting final classification outcomes and sacrificing reproducibility.

Other studies have extensively investigated the relationship between neural signals and brain anatomy in the mouse hippocampus, demonstrating remarkable accuracy in discriminating hippocampal subregions (Berényi et al., 2014; Angotzi et al., 2025; Maslarova et al., 2025; Paleologos et al., 2025; Khanzada et al., 2026). However, these methods rely on prior knowledge of the functional and anatomical organization of the target structure and are therefore difficult to generalize or rapidly adapt to other brain regions. Other approaches based on different electrode neurotechnologies and electrophysiological signals have also been proposed for anatomical localization, including surface ECoG grids (Branco et al., 2018) and DBS electrodes (Miyagi et al., 2009; Oxenford et al., 2022). The main features of these methods are summarized in Supplementary Table 1.

In this work, we introduce LFP-LOC, a novel tool for anatomical localization of neural probes that (i) can be seamlessly applied across different brain regions, probe technologies, electrode layouts, and experimental conditions, (ii) requires short data durations and low computational overhead and, (iii) yields interpretable results, and (iv) automatically refines probe location within a neighborhood of user-specified stereotactic coordinates.

LFP-LOC builds on the observation that different brain regions are characterized by distinct spectral signatures in low frequency activity (Mantini et al., 2007; Chen et al., 2008; Groppe et al., 2013; Wang et al., 2025). Specifically, we compute power in the canonical LFP bands and cluster electrodes that exhibit similar spectral profiles. We then compare the resulting clusters with known brain anatomy and observe a strong correspondence between LFP signatures and anatomical boundaries. The high spatial resolution afforded by CMOS-based neural probes proved essential for reliable clustering and for resolving fine-grained anatomical features, particularly in regions with complex substructures such as the hippocampus.

We believe that LFP-LOC represents an important step toward intraoperative validation and refinement of probe implant location, substantially reducing the reliance on *post-hoc* histological analysis. In addition, the inferred anatomical context can be used to inform downstream analyses, such as cell-type identification performed with complementary tools [e.g., CellExplorer (Petersen et al., 2021)]. Beyond allowing to identify probe location, our framework offers a means to systematically investigate how distinct brain regions operate in LFP spectral bands. This capability is especially relevant for bridging the gap between animal studies and clinical research, where spectral power is typically assessed using noninvasive techniques with low spatial resolution.

## 2 Materials and methods

### 2.1 Electrophysiology datasets and probe layouts

To evaluate the robustness and generalizability of LFP-LOC, we analyzed a set of five in-vivo LFP recordings spanning multiple brain regions, probe layouts, technologies, and experimental conditions. The datasets included three brain regions (hippocampus, *n* = 2; visual cortex, *n* = 2; medial prefrontal cortex, *n* = 1), two probe technologies (SiNAPS, Corticale, Italy, *n* = 4; Neuropixels 2.0, *n* = 1), three electrode layouts (SiNAPS single-shank, *n* = 2; SiNAPS eight-shank, *n* = 2; Neuropixels 2.0 four-shank, *n* = 1), and three experimental conditions (anesthetized, *n* = 2; head-fixed, *n* = 2; freely moving, *n* = 1).

**Figure 1:**
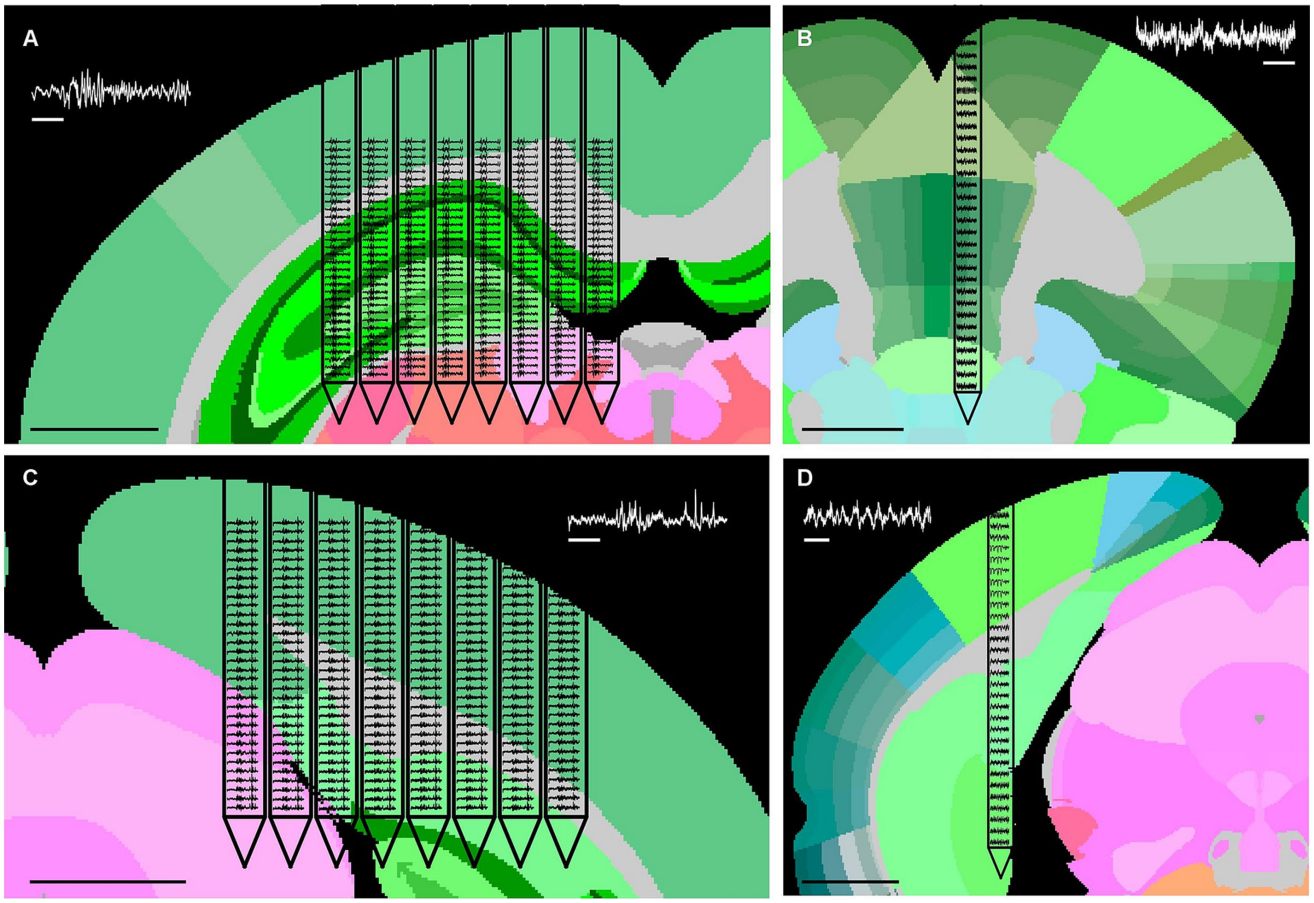
Representative datasets acquired with high-density SiNAPS probes. Representative examples of *in vivo* LFP recordings acquired with high-density SiNAPS probes across distinct brain regions and probe configurations. **(A)** Eight-shank 1,024 channels probe implanted in the hippocampus. **(B)** Single-shank 256 channels probe implanted in the medial prefrontal cortex. **(C)** Eight-shank 1,024 channels probe implanted in the visual cortex. **(D)** Single-shank 256 channels probe implanted in the visual cortex. For each dataset, 5 s of LFP activity (1–300 Hz) are shown across the full probe span and overlaid on the corresponding anatomical context. White scale bars, 1 s; black scale bars, 1 mm.

- Eight-shank SiNAPS probe (1,024 channels) implanted in the hippocampus, head-fixed animal (Figure 1A; AP: −2.3 mm, ML: 2.6 mm, DV: −2.8 mm).
- Single-shank SiNAPS probe (256 channels) implanted in the medial prefrontal cortex, freely moving animal (Figure 1B; AP: 1.8 mm, ML: −0.3 mm, DV: −4.9 mm).
- Eight-shank SiNAPS probe (1,024 channels) implanted in the visual cortex, anesthetized animal (Figure 1C; AP: −3.5 mm, ML: −1.3 mm, DV: −2.3 mm).
- Single-shank SiNAPS probe (256 channels) implanted in the visual cortex, anesthetized animal (Figure 1D; AP: −3.8 mm, ML: 2.7 mm, DV: −4.2 mm).
- Two side-by-side Neuropixels 2.0 four-shank probes (2 × 384 channels) implanted in the hippocampus (Paleologos et al., 2025), head-fixed animal (probe 1 coordinates AP: −2 mm, ML: −0.4 mm, DV: −4.2 mm, probe 2 coordinates AP: −2 mm, ML: −1.4 mm, DV: −4 mm).

Figure 1 displays examples of the SiNAPS probes datasets used in this work. The layout features of the devices used in this work are summarized in Table 1.

**Table 1:** Summary table of device layout features.

Device type	N shanks	N columns	Shank pitch (μm)	Shank Width (μm)	Vertical pitch (μm)	Horizontal pitch (μm)
Single-shank SiNAPS	1	2	-	88	30	27
Eight-shank SiNAPS	8	2	300	88	30	27
NeuroPixel 2.0	4	2, 1 selected per shank	250	70	15	32

### 2.2 Step 1 - data processing and spectral power estimation

**Figure 2:**
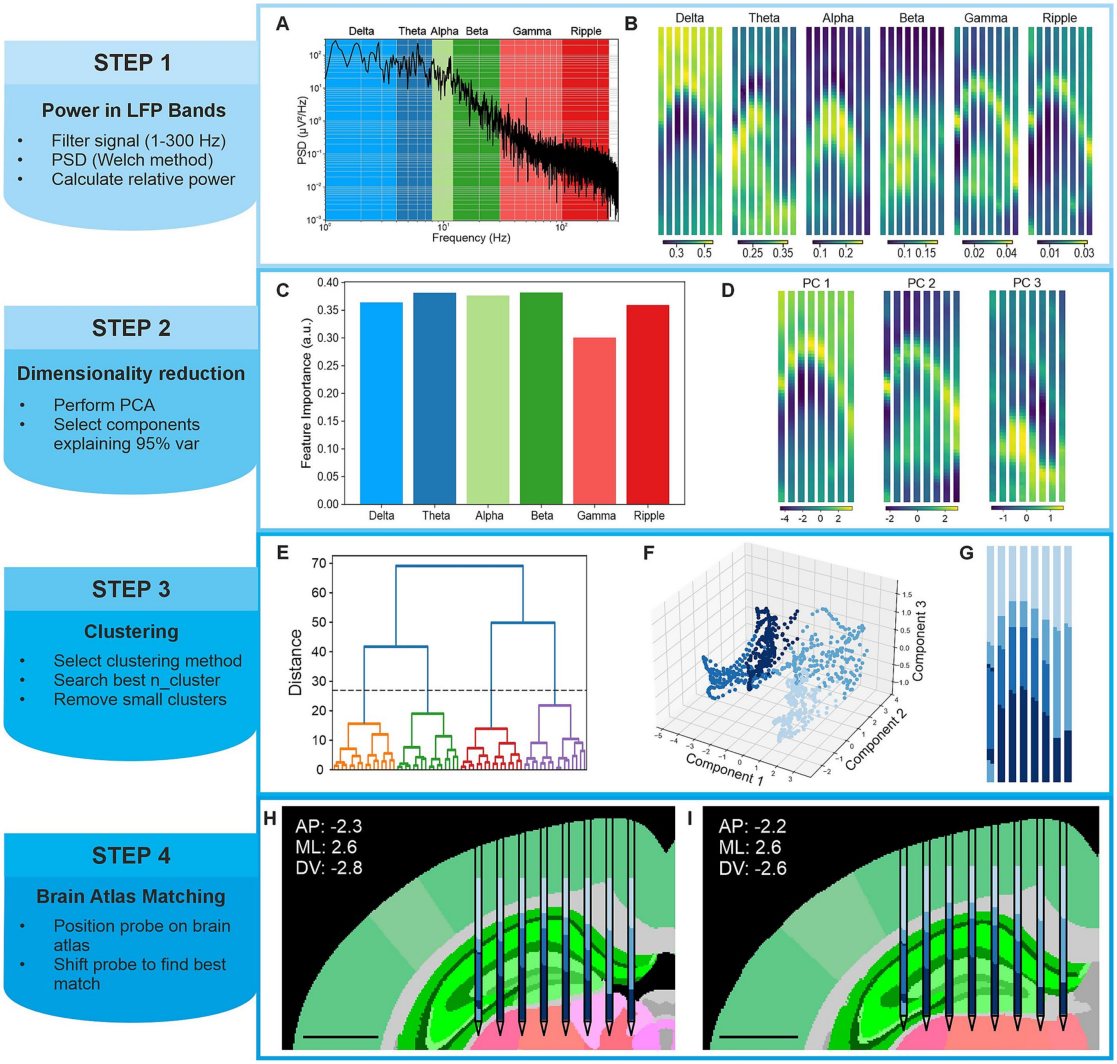
LFP power–based anatomical localization pipeline. Overview of the computational workflow used to infer anatomical structure from spatial distributions of LFP spectral power, illustrated for an eight-shank 1,024 channels hippocampal recording. **(A)** Representative power spectral density (PSD) from a single electrode, highlighting canonical LFP frequency bands (delta, theta, alpha, beta, gamma) and the ripple band (normalized power). **(B)** Spatial maps of normalized band-limited LFP power after local outlier removal and spatial smoothing, projected onto probe geometry. **(C)** Relative contribution of individual frequency bands to the reduced feature space obtained by principal component analysis (PCA). **(D)** Spatial distribution of the retained principal components (arbitrary units) across the probe. **(E)** Hierarchical clustering dendrogram computed from PCA-reduced features, with the selected cut distance indicated. **(F)** Electrode clusters in reduced feature space. **(G)** Final cluster assignments projected onto probe geometry. **(H)** Initial alignment of clusters to the brain atlas based on user-defined stereotactic coordinates. **(I)** Refined probe position after atlas-based optimization to maximize correspondence between clusters and anatomical regions. Scale bars, 1 mm.

The sequence of steps constituting the LFP-LOC algorithm is summarized in Figure 2.

All analyses were performed using custom Python scripts, available at this GitHub repository: https://github.com/raffaele222/LFP-LOC. For each experiment, a user-defined segment of the electrophysiological recording was selected and loaded for analysis. For the SiNAPS recordings, we used 30 s of data, while for the NeuroPixel recording we used 100 s of data. Raw signals were band-pass filtered in the LFP band (1–300 Hz). Recordings acquired with SiNAPS probes were downsampled to 1,250 Hz to match the sampling rate of Neuropixels LFP data and to reduce computational overhead. In addition, the Python implementation includes an optional common average reference (CAR) preprocessing step that can be applied before PSD estimation when shared-channel contamination is suspected. We do not recommend CAR as a default step, because in recordings with good signal quality and no substantial common artifacts it may remove biologically meaningful components shared across neighboring electrodes and promote over-splitting of otherwise coherent clusters.

Power spectral density (PSD) estimates were computed independently for each electrode using the Welch method and are expressed in linear power units (μV^2^/Hz). PSDs were calculated with a window length of 8,192 samples (corresponding to the closest power of two to 6 s at 1,250 Hz), a Hann window, and 50% overlap between consecutive windows. Spectral power was then obtained by integrating the PSD within the canonical LFP frequency bands (delta: 1–4 Hz, theta: 4–8 Hz, alpha: 8–12 Hz, beta: 12–30 Hz, gamma: 30–100 Hz) and the ripple band (100–250 Hz). To account for variability in overall signal amplitude across electrodes, band-specific power values were normalized by the total power in the LFP band (1–300 Hz).

To remove electrodes exhibiting anomalous spectral profiles, we applied a local outlier detection procedure independently to each shank. For each frequency band, electrodes were evaluated within adjacent windows of 20 neighboring electrodes, and those with an absolute z-score greater than 2.5 were excluded. The choice of z-score threshold reflects a trade-off between removing extreme outliers that could bias subsequent spatial smoothing and preserving electrodes that may exhibit genuine anatomical differences in spectral power. A threshold of 2.5 was selected as a conservative criterion, corresponding to approximately 1% probability under a normal distribution, thereby targeting only the most pronounced deviations. Lower thresholds may lead to the removal of physiologically relevant signals, whereas higher thresholds may allow large outliers to influence the moving average filter.

In the datasets analyzed, the 20-electrode window corresponds to a vertical span of approximately 300 μm for both probe types, based on their respective electrode pitches and configurations. This window size was chosen to provide sufficient local context for robust estimation of statistical properties (mean and variance) while preserving spatial specificity along the probe. Although spanning sharp anatomical boundaries may in principle lead to misclassification of valid signal transitions, this risk is mitigated by the conservative z-score threshold, which targets only extreme deviations. Both the window size and z-score threshold can be adjusted depending on probe geometry and dataset characteristics.

Following outlier removal, spatial smoothing was applied to the remaining features to reduce local variability. Specifically, a moving average filter with a window size of 8 electrodes was applied independently along each shank, corresponding to an effective vertical smoothing length of 120 μm. This window size was selected to improve spatial homogeneity of the extracted features while preserving high spatial resolution. The choice of smoothing window length can be further refined by the user according to the spatial distribution of power features in the dataset under investigation. If a fine-grained distribution of power features is observed, a shorter smoothing window will better preserve spatial resolution, whereas a larger smoothing window may be more appropriate when substantial local variability occurs within otherwise homogeneous regions.

### 2.3 Steps 2 and 3 - dimensionality reduction and clustering

Dimensionality reduction was performed using principal component analysis (PCA) to capture the dominant structure of the spectral power features. Prior to PCA, features were standardized (z-scored) using the StandardScaler module from sklearn.preprocessing. PCA was then computed using the PCA implementation in sklearn.decomposition. The number of retained principal components was selected such that the cumulative explained variance exceeded 95%, allowing the dimensionality of the reduced feature space to adapt automatically to each dataset.

To assess the relative contribution of individual frequency bands to the resulting principal components, feature relevance was quantified by multiplying the absolute loading of each power band onto each principal component by the variance explained by that component and summing across retained components. This metric provided an interpretable estimate of the contribution of each spectral band to the reduced representation.

Unsupervised clustering was subsequently performed on the retained principal components using hierarchical agglomerative clustering (AgglomerativeClustering, sklearn.cluster). The number of clusters was varied between 3 and 12, and clustering performance was evaluated for each solution using the silhouette score (silhouette_score, sklearn.metrics). The number of clusters yielding the highest silhouette score was selected, and each electrode was assigned to the corresponding cluster. In addition to hierarchical agglomerative clustering, we evaluated alternative clustering approaches, including k-means and HDBSCAN, to assess the robustness of the results to the choice of algorithm. All methods were systematically applied to all datasets using the same input features. For k-means, the number of clusters was varied within the same range as for hierarchical clustering (3–12), and the optimal solution was selected based on the silhouette score. HDBSCAN was applied with parameters selected to reflect the spatial scale of the data. Across datasets, hierarchical clustering and k-means produced comparable and spatially coherent cluster assignments, whereas HDBSCAN tended to yield smaller and less anatomically consistent clusters. Based on these observations, hierarchical clustering was selected as the primary method for all subsequent analyses.

### 2.4 Brain atlas selection and coordinates convention

Anatomical reference data were accessed using the *brainglobe_atlasapi* (Claudi et al., 2020) Python package, which enables programmatic loading and manipulation of labeled mouse brain atlases within our Python-based analysis pipeline. This framework allowed direct comparison between electrode clusters derived from LFP spectral features and known anatomical structures.

All anatomical coordinates are defined relative to bregma, following the coordinate system implemented in *brainglobe_atlasapi*. In this framework, bregma corresponds to the atlas coordinates AP = 5.4 mm, ML = 0 mm, and DV = 0 mm. Positive AP values indicate positions anterior (rostral) to bregma, whereas negative AP values correspond to more posterior locations. ML coordinates are defined such that positive values indicate positions in the left hemisphere and negative values indicate positions in the right hemisphere. DV coordinates are defined with increasing depth represented by more negative values, such that increasingly negative DV values correspond to deeper probe implantations. All stereotactic coordinates reported in this study follow this convention.

Among the atlases available through *brainglobe_atlasapi*, we selected the *kim_mouse_isotropic_20um* (Chon et al., 2019) atlas, as it provides a favorable balance between spatial resolution and completeness of anatomical annotation. In contrast to higher-resolution atlases with more limited labeling, this atlas offers comprehensive regional coverage across the mouse brain, which is essential for assigning electrode clusters to anatomical regions and for evaluating correspondence between electrophysiological features and anatomy.

Importantly, the atlas includes detailed subdivisions within large structures, such as cortical areas and hippocampal strata. For datasets acquired with multi-shank (eight-shank SiNAPS and NeuroPixel 2.0) probes, this fine-grained anatomical parcellation proved overly specific for the spatial scale represented by the electrophysiological clusters. In these cases, clusters typically corresponded to broader anatomical entities, such as the cortex as a whole or individual hippocampal layers (e.g., stratum oriens, radiatum, and lacunosum-moleculare), rather than to smaller subregions. To avoid spurious mismatches during atlas-based localization, we therefore modified the atlas data by merging all cortical subregions into a single cortical label and by consolidating hippocampal strata into unified layer-level labels. The code used to perform this atlas relabeling and the resulting NumPy arrays (relabelled_atlas.npy and final_rgb_map.npy) are provided in the shared GitHub repository.

For single-shank probe datasets, the original atlas labeling was retained. In these experiments, a single shank traverses cortical layers along the dorsoventral axis, and clusters may reflect finer anatomical subdivisions, as observed in the medial prefrontal cortex dataset. In this context, preserving the native atlas granularity did not adversely affect probe repositioning and allowed more precise anatomical assignment of electrode clusters.

### 2.5 Step 4 - brain atlas matching

The probe was initially positioned within the brain atlas according to the stereotactic coordinates targeted during implantation and provided by the user. The AP coordinate was used to select the corresponding coronal atlas slice, and the probe was placed by aligning the tip of the first shank to the specified ML and dorsoventral DV coordinates. Electrode positions, expressed in micrometers, were converted to atlas voxel coordinates dividing by the spatial resolution of the atlas. Clustered electrodes and the probe outline were then overlaid onto the atlas, providing an initial qualitative assessment of correspondence between electrophysiological clusters and anatomical structures.

To refine probe placement, we implemented a local search algorithm under the assumption that the initial stereotactic estimate was close to the true implant location. First, each electrode cluster was assigned to an anatomical region by identifying the most frequently occurring atlas label among the voxels corresponding to the electrodes within that cluster (mode assignment).

Probe position was then systematically perturbed within a local neighborhood of the initial estimate. Specifically, the probe was shifted across adjacent coronal sections along the AP axis (eight slices in both anterior and posterior directions) and, within each slice, translated along the ML and DV axes with a maximum displacement of ±600 μm. For every candidate position, we computed the number of electrodes whose atlas labels matched the anatomical region assigned to their respective cluster. This refinement procedure assumes that the initial targeting error is within this spatial range. If the true probe displacement exceeds these bounds, the algorithm will return the best match within the defined search space, which may not correspond to the correct anatomical location. While the search range can be extended by the user, increasing it introduces the risk of identifying spurious matches in anatomically unrelated regions. As a result, there is a trade-off between robustness to initial targeting errors and the specificity of the refinement.

The combination of AP, ML, and DV offsets maximizing the number of electrodes assigned to their expected anatomical region was selected as the refined probe position. The updated probe placement was subsequently displayed on the atlas for visualization and validation.

### 2.6 Spatial downsampling of electrophysiological data

To visualize and quantify the impact of spatial subsampling on the proposed processing pipeline, we evaluated band-limited power features across the full geometrical extent of the probe while progressively reducing the number of electrodes used for feature extraction. Spatial subsampling was controlled using a decimation factor (DF), defined as the ratio between the original electrode density and the retained electrode density along the probe. A DF of 1 corresponds to the full-resolution configuration (all electrodes retained), while higher values correspond to progressively sparser sampling. For each DF, electrodes were selected by uniformly subsampling the probe layout. For example, a DF of 2 corresponds to retaining every second electrode (or a single column in multi-column configurations), while a DF of 4 corresponds to retaining one electrode every two rows, effectively increasing the vertical pitch by a factor of two. Higher DFs were defined analogously, resulting in progressively coarser spatial sampling.

For each decimation condition, power features were first computed for the selected subset of electrodes. To generate continuous spatial maps of the band-specific power features, rather than values defined only at electrode locations, we constructed a uniformly spaced grid spanning the probe geometry with an arbitrary (user-defined) spatial resolution. Feature values at non-sampled grid points were then estimated by linear interpolation of the values from the selected electrodes. This spatially enhanced representation enabled direct visualization of how decreasing electrode density affects the apparent spatial resolution and smoothness of the power features.

Clustering was subsequently performed on the interpolated feature maps to assess the effect of spatial subsampling on cluster structure. To quantify similarity between clustering solutions obtained with different DFs, we computed a cluster matching score using the full-resolution clustering as a reference. For each DF, one of the reference clusters was assigned to the subsampled clusters which best matched it (largest number of overlapping grid points).

The matching score was then defined as the fraction of grid points assigned to the same cluster as in the full-resolution condition, normalized by the total number of grid points. This metric provided a direct and interpretable measure of how spatial subsampling degrades the ability of the pipeline to recover anatomically consistent clustering patterns.

### 2.7 Feature convergence

To determine the amount of data required for stable estimation of band-limited spectral power, we computed power features using progressively longer segments of each recording. For each recording duration, spatial power maps were generated for all frequency bands and compared to the corresponding maps obtained using the full recording length (300 s), which served as the reference.

To ensure that convergence estimates were not dominated by low-frequency bands with higher absolute power, we quantified differences using a normalized metric. Specifically, for each frequency band, the average difference (pixel by pixel) between the power map computed from a partial recording and the reference map was divided by the standard deviation of the pixels in the reference power map. This normalization equalized the contribution of all frequency bands to the convergence metric, allowing fair comparison across spectral ranges.

To identify the recording duration at which power estimates stabilized, we defined a plateau threshold based on the evolution of the normalized difference. The plateau value was computed as the mean of the final six time points, corresponding to a region where the difference had visually reached a stable asymptote, plus three standard deviations. The time to convergence was then defined as the earliest recording duration at which the normalized difference fell below this threshold.

Although the plateau threshold is heuristic, it provides a consistent and interpretable reference for estimating the recording duration required to obtain reliable spectral power features across experimental conditions.

## 3 Results

To evaluate and validate LFP-LOC, we applied it to a diverse set of in-vivo electrophysiological recordings from different laboratories acquired across multiple brain regions, probe technologies, electrode layouts and experimental conditions. This dataset diversity was chosen to assess the robustness and generalizability of the proposed method.

Figure 1 illustrates representative examples from the SiNAPS probes (Angotzi et al., 2019, 2025; Ribeiro et al., 2022) datasets used in this work, showing 5 s of raw LFP traces superimposed on the corresponding brain anatomy.

These recordings span both cortical and subcortical structures and include probes with varying shank counts, geometries, and fabrication technologies. All datasets were acquired using high-density probes, a requirement we found to be critical for reliable identification of anatomical boundaries based on spatially resolved LFP power.

Figure 2 provides an overview of the main steps of the proposed algorithm applied to the case of the 8-shank hippocampus recording, illustrating the processing pipeline from raw LFP signals to the identification of electrode clusters that correspond to distinct anatomical regions.

### 3.1 Spatial LFP band-power maps provide robust localization features

Prior to spectral analysis, raw electrophysiological signals were band-pass filtered in the LFP band (1–300 Hz) and downsampled from the original sampling frequency (i.e., 20 kHz for SiNAPS data) to 1,250 Hz to reduce computational load while preserving relevant low-frequency content. PSDs were then computed independently for each electrode using Welch’s method, using a moving window of around 6 s. The choice of window length reflects a trade-off between spectral resolution at low frequencies and temporal resolution required for rapid convergence of PSD estimate within a short recording interval, which is crucial in the perspective of online intraoperative implementation. Empirically, a six second window provided a robust compromise across datasets, although this parameter can be adapted to specific experimental requirements.

Figure 2A illustrates a representative power spectrogram computed for a single electrode. From the PSDs, spectral power was integrated within the canonical LFP frequency bands (delta, theta, alpha, beta, and gamma) and within the ripple band (100–250 Hz). Band-specific power values were then mapped onto the probe geometry, as shown in Supplementary Figure 1A, displaying clear spatial organization across the probe geometry.

To provide an overview of the variability in LFP power across datasets, we quantified the distribution of power spectral density values in the 1–300 Hz range. To minimize the influence of outliers, we report the 1st and 99th percentiles of the PSD values, expressed both in linear units (μV^2^/Hz) and in decibels. Across datasets, the 1st percentile ranged from approximately $10^{-9}$ (electrodes outside of brain tissue) to $10^{3}$ μV^2^/Hz, while the 99th percentile ranged from approximately $10^{3}$ to $5\times 10^{4}$ μV^2^/Hz. In decibel units, this corresponds to a range from approximately −87 dB to 47 dB. These results highlight the substantial variability in absolute LFP power across recordings and further motivate the use of normalized band-power features in our analysis. We have reported the individual ranges of the SiNAPS datasets used in this work in Supplementary Table 2.

To improve spatial consistency of the extracted features, electrodes exhibiting power values that markedly deviated from those of their neighbors were identified and removed using a local outlier detection procedure (Supplementary Figure 1B). The outlier removal procedure resulted in the exclusion of a small fraction of electrodes (2.03 ± 0.61% across datasets), with an average of 0.39 ± 0.13 electrodes removed per 20-channel window. The proportion of electrodes removed across individual datasets is reported in Supplementary Table 3. Subsequently, a moving average filter was applied across neighboring electrodes to smoothen spatial fluctuations, yielding the refined power maps shown in Figure 2B and Supplementary Figure 2. These preprocessing steps were essential to enhance feature homogeneity and stability prior to dimensionality reduction and clustering.

### 3.2 Unsupervised clustering of band-power recovers contiguous anatomical domains

To distill the most informative structure from the extracted spectral power features, we applied principal component analysis (PCA). Across all experimental conditions, the contributions of individual frequency bands to the principal components were comparable (Figure 2C; Supplementary Figures 3A–C), indicating that the canonical LFP frequency bands carry similarly informative content for characterizing electrode-specific spectral signatures. This observation suggests that no single frequency band dominates the anatomical organization captured by LFP-LOC. Rather, the spectral signature of a given brain region appears to arise from a combination of contributions across multiple frequency bands. This is consistent with the notion that LFP activity reflects a superposition of neural processes, including synaptic inputs and population dynamics, which span a broad frequency range and are not uniquely associated with specific anatomical regions. As a result, individual frequency bands may not be sufficient on their own to discriminate brain areas, whereas their combined representation provides a more informative and robust characterization of local electrophysiological structure. This finding further supports our choice of leveraging multi-band power features for anatomical localization. An example of the feature loadings from the eight-shank hippocampus recording is shown in Supplementary Figure 3D. The spatial distribution of the resulting principal components projected onto the probe geometry is shown in Figure 2D.

Electrodes were subsequently grouped based on their reduced feature representations using unsupervised clustering. We implemented and evaluated multiple clustering strategies, including hierarchical, k-means, and HDBSCAN, across all datasets. Hierarchical clustering and k-means consistently produced comparable and anatomically coherent cluster assignments, whereas HDBSCAN resulted in smaller and less consistent clusters. A representative example is shown in Supplementary Figure 4 for the hippocampus and visual cortex datasets, with similar trends observed across other datasets. Figure 2E shows a representative dendrogram used for hierarchical clustering, with the selected cut distance indicated by a dashed line.

The number of clusters was constrained to vary between three and twelve, a range that consistently captured meaningful anatomical subdivisions without over-segmentation, and the solution yielding the highest silhouette score was selected (Supplementary Figure 3E). The final cluster assignment, displayed on the probe geometry in Figure 2G, reveals spatially contiguous and well-organized electrode groups. Notably, this spatial coherence emerged despite the absence of explicit spatial information during both dimensionality reduction and clustering underscoring the strong relationship between LFP spectral power and underlying brain anatomy. While spatial information was implicitly used for local outlier removal and smoothing, the power distribution exhibited pronounced spatial dependence even before these preprocessing steps were applied, as shown in Supplementary Figure 1A.

### 3.3 Atlas-based refinement improves electrode cluster–anatomy agreement

The probe geometry with the identified electrode clusters was subsequently overlaid onto an anatomical model of the mouse brain at the stereotactic coordinates targeted during the experiment. Atlas data were accessed using the *brainglobe_atlasapi* (Claudi et al., 2020) Python package, and the *kim_mouse_isotropic_20um* (Chon et al., 2019) atlas was selected because it combines high spatial resolution with extensive regional annotation, in contrast to other available atlases that offer higher resolution but limited labeling.

Integrating atlas information through a readily accessible software framework facilitates systematic comparison between electrophysiological features and brain anatomy. To our knowledge, this approach is not widely adopted for probe localization and could be readily extended to validate implant position using alternative or complementary signal-derived features.

The probe geometry was first scaled to match atlas dimensions and subsequently positioned at the targeted stereotactic coordinates. The localization procedure assumes that the implant is close to the selected location and is designed to refine probe position within a local neighborhood. To refine probe placement, the probe was then systematically shifted across adjacent coronal planes, as well as along the mediolateral and dorsoventral axes. The extent of this search can be adapted to the experimental context. For each candidate position, the number of electrodes assigned to the appropriate anatomical region was computed.

**Figure 3:**
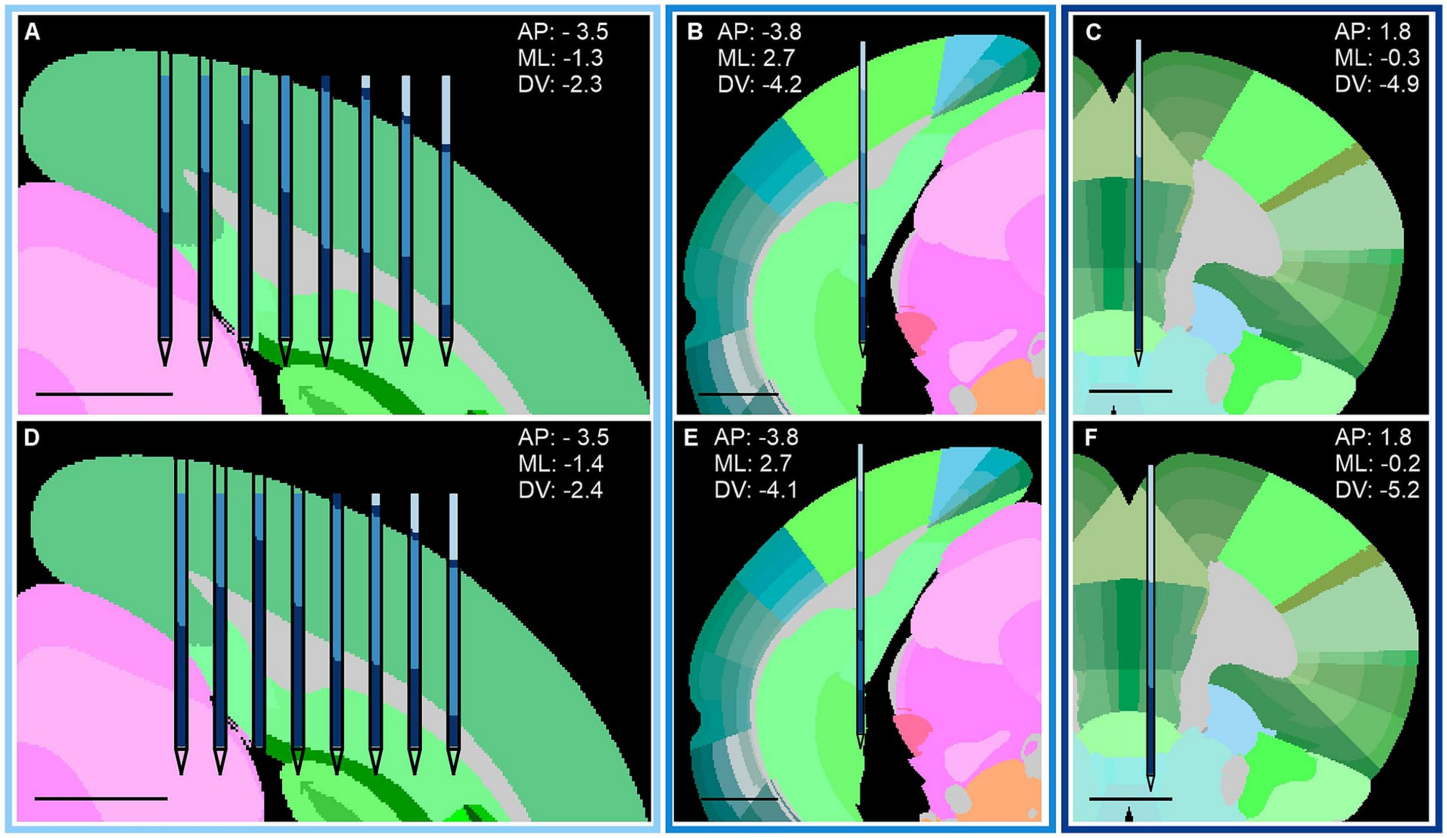
Atlas-based refinement of probe position across brain regions. Probe localization before and after atlas-based refinement for different brain regions and probe configurations. **(A,D)** Visual cortex, eight-shank probe. **(B,E)** Visual cortex, single-shank probe. **(C,F)** Medial prefrontal cortex, single-shank probe. Top panels show probe placement based on stereotactic coordinates; bottom panels show refined positions obtained by maximizing agreement between LFP-derived clusters and atlas annotations. Scale bars, 1 mm.

Supplementary Figure 5A shows representative examples of the resulting matrices across different coronal planes. The configuration maximizing the number of electrodes assigned to anatomically consistent regions is indicated by a red marker and selected as the refined implant location. Figures 2H,I, 3 illustrate the correspondence between electrode clusters and brain anatomy before and after position refinement, demonstrating improved alignment between clusters and anatomical regions across experimental settings. Supplementary Figure 5B displays the percentage of clusters in the appropriate region before and after adjustment, with an average increase of 8.2% ± 6.6% across datasets. Individual clusters can span multiple anatomical regions, while a single anatomical region may be partitioned into several clusters. This lack of a one-to-one correspondence between electrode clusters and anatomical regions, combined with inherent inter-individual anatomical variability, explains why the overall proportion of correctly assigned electrodes remains relatively modest (<65%) even after realignment. Nevertheless, probe position refinement leverages multiple consistent correspondences between clusters and anatomical structures across the active area span, thereby improving robustness despite imperfect electrode-to-region assignments.

The performance of the algorithm depends on the accuracy of the initial stereotactic coordinates provided by the user and large initial targeting errors may limit refinement accuracy. Nevertheless, the approach enables rapid visual assessment of whether the implant is correctly placed or substantially misplaced and provides a practical means to refine probe location within a local neighborhood. Although atlas-based localization is inherently constrained by inter-animal anatomical variability, we expect LFP-LOC to be more reliable and substantially less labor-intensive than *post hoc* immunohistochemical reconstruction for validating probe placement across experiments.

### 3.4 High electrode density is critical for resolving anatomical boundaries

Given that the spatial reach of LFP signals is on the millimeter scale (Schwartz et al., 2006), high-spatial-resolution recordings are typically regarded as oversampling, with limited added value. To address this question, and specifically to assess how electrode density influences feature extraction and clustering performance, we systematically performed spatial downsampling by computing power features from progressively sparser subsets of electrodes. To facilitate visual comparison across conditions, power features obtained from the selected electrodes were linearly interpolated to generate continuous feature maps spanning the geometrical extent of the probe.

**Figure 4:**
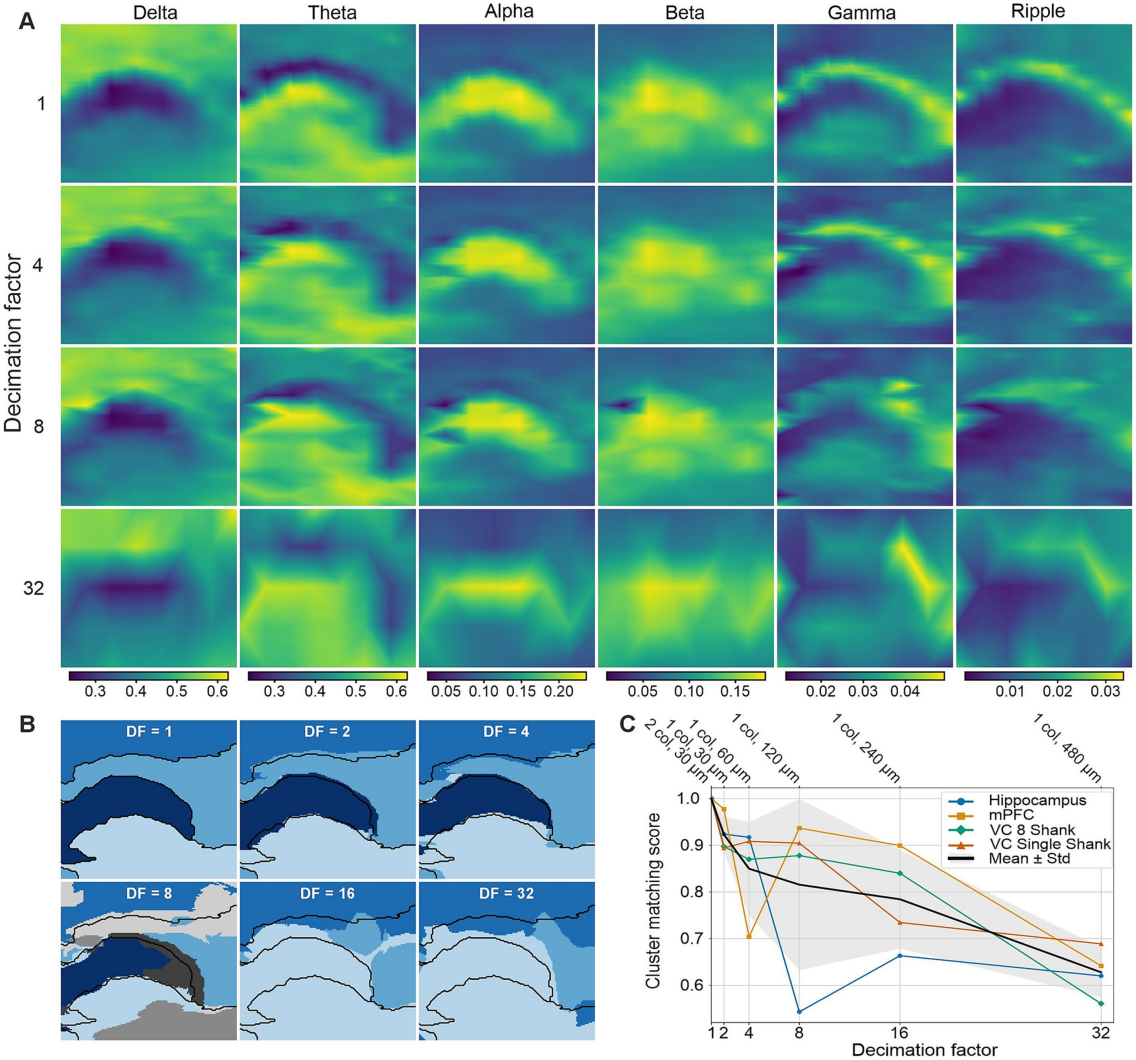
Effect of spatial sampling density on LFP power-based anatomical clustering. **(A)** Spatial distributions of band-limited LFP power (normalized power) as a function of the spatial decimation factor (DF) in the 8-shank hippocampus dataset. Increasing DF corresponds to progressively sparser electrode sampling; power values are linearly interpolated between retained electrodes to convey more clearly the effect of spatial downsampling. **(B)** Cluster assignments obtained for each DF. Black outlines indicate clusters derived from full-resolution data (DF = 1). **(C)** Cluster matching score as a function of DF across datasets. The number of columns and the vertical pitch corresponding to each DF are indicated on the secondary x axis (top) to facilitate comparisons with other probe technologies. Colored curves represent individual experiments; the black curve and gray shaded area indicate mean ± s.d. across datasets.

Figure 4A shows representative feature maps obtained for the different DFs tested. A DF of 2 corresponds to retaining a single electrode column, whereas a factor of 4 corresponds to selecting one electrode every two rows, thereby doubling the effective vertical pitch. Higher DFs were defined analogously.

Clustering was then applied to the interpolated feature maps corresponding to each DF (Figure 4B). For each DF greater than one, cluster assignments were compared to those obtained at full spatial resolution. Figure 4C summarizes the cluster matching scores across experimental conditions as a function of DF. On average, the matching score decreased monotonically with increasing decimation, highlighting the importance of dense electrode coverage for accurately resolving the anatomical distribution of LFP spectral power. Notably, the impact of spatial downsampling varied across brain regions. In the hippocampus, a DF of 8, corresponding to a vertical pitch of 120 μm, already led to a pronounced reduction in matching score (below 0.6). In contrast, in the visual cortex, matching scores remained above 0.8 and cluster assignments remained visually reliable up to a DF of 16 (Supplementary Figure 6).

These differences likely reflect region-specific anatomical organization. In the hippocampus, fine-grained laminar and subregional structures require high spatial sampling density for reliable discrimination, whereas in the visual cortex, larger-scale anatomical features can be resolved with coarser spatial sampling.

### 3.5 Short recordings are sufficient for stable power estimates

**Figure 5:**
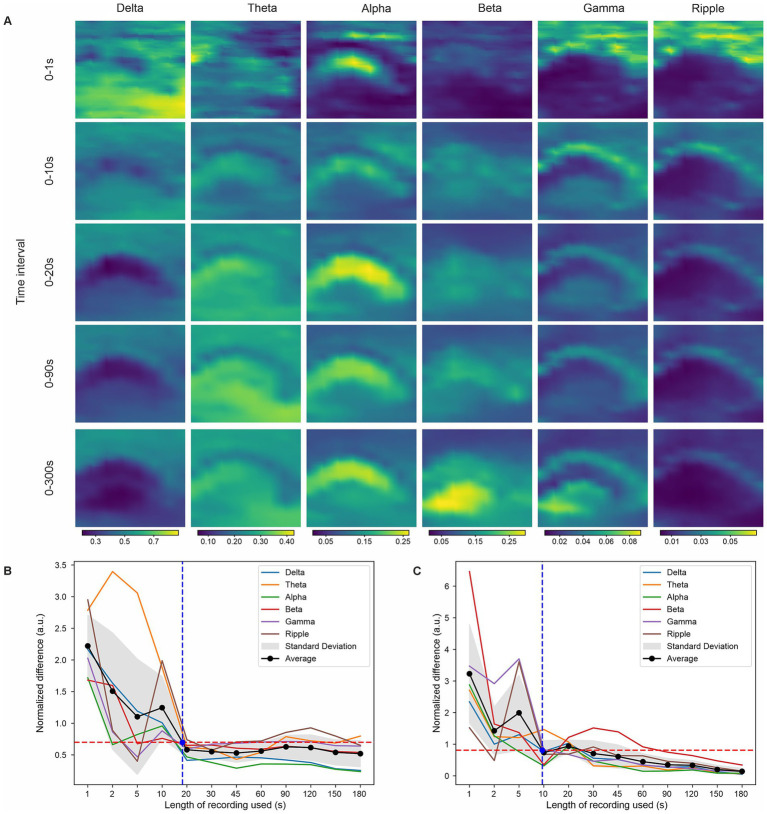
Convergence of spectral power estimates with recording duration. **(A)** Representative spatial maps of band-specific LFP power (normalized power) computed from progressively longer recording durations for the 8-shank hippocampus dataset. **(B,C)** Normalized difference between power features estimated from partial recordings and those computed from the full 300 s dataset, shown for the eight-shank hippocampus (head-fixed) **(B)** and eight-shank visual cortex (anesthetized) **(C)** recordings. Colored curves indicate individual frequency bands; black curves indicate the average across bands. Shaded regions denote s.d. The horizontal red dashed line marks the convergence plateau threshold; the vertical blue dashed line indicates the minimum recording duration required for stable feature estimation.

To determine the duration of data required for reliable estimation of spectral power features, we computed power spectral densities using progressively longer segments of each recording and compared the resulting features with those obtained from the full recording duration (300 s). Figure 5A shows representative spatial maps of band-specific power computed from selected time intervals in the hippocampus dataset, while Supplementary Figure 7 shows the same plots for the 8-shank visual cortex dataset.

Feature convergence was quantified by calculating the normalized difference between power features extracted from partial recordings and those derived from the full dataset. However, this analysis is based on progressively longer contiguous segments of the recording and does not assess whether any arbitrary time window of equivalent duration would yield comparable feature estimates. Figures 5B,C show the normalized differences as a function of recording duration for the eight-shank hippocampus and visual cortex datasets, respectively. Across experimental conditions, power estimates consistently converged to a stable plateau within approximately 10–20 s of data, with minor variations depending on brain region and recording characteristics.

Importantly, in awake recordings, where LFP activity can exhibit state-dependent fluctuations, we observed that spatial patterns of band-limited power emerge rapidly and stabilize within the same 10–20 s timescale. This indicates that transient, state-dependent components are effectively averaged out during spectral estimation, while stable, region-specific features dominate the representation used for clustering.

This rapid convergence demonstrates that robust spectral power features can be obtained from relatively short recording windows and is partly attributable to the appropriate selection of Welch window parameters used for power spectral density estimation. In practice, this would allow experimenters to validate and refine probe placement intraoperatively using brief recordings (on the order of 20–30 s), either by running the analysis immediately after acquisition or by integrating the pipeline directly into the recording software.

The convergence metric assigns equal weight to all frequency bands to avoid dominance by bands with higher absolute power. Although lower-frequency bands might be expected to require longer recording durations for stable estimation, we observed similar convergence dynamics across bands. Some residual variability is present after the plateau, reflecting intrinsic fluctuations in neural activity, particularly in awake recordings. While longer recordings may further improve stability, the observed convergence within 10–20 s provides a practical lower bound that balances estimation reliability with acquisition time.

### 3.6 Validation on Neuropixels 2.0 dataset

We applied the same processing pipeline to a high-density electrophysiology dataset acquired using Neuropixels 2.0 probes (Paleologos et al., 2025). Because each Neuropixels 2.0 probe records from a maximum of 384 channels simultaneously and spans a limited lateral extent (~1 mm), in this study authors used two probes that were implanted side by side to achieve sufficient spatial coverage of the hippocampus. Further, to maximize depth coverage for Neuropixels 2.0 recordings, a single column of electrodes was selected from each shank.

**Figure 6:**
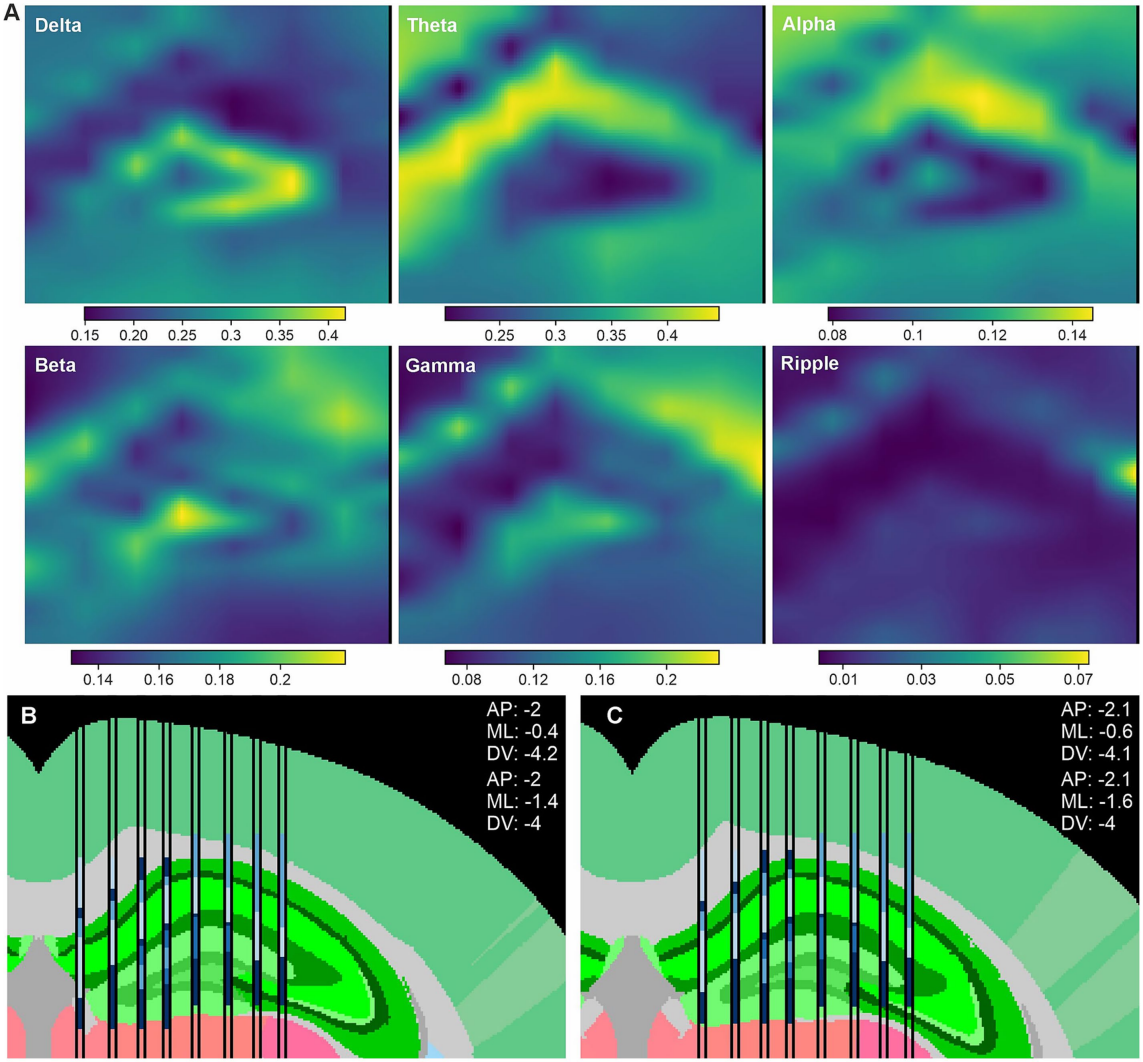
Application to Neuropixels 2.0 recordings. Anatomical localization using two Neuropixels 2.0 probes implanted side by side in the hippocampus. **(A)** Spatial distribution of band-limited LFP power (normalized power) across the combined probe geometry (normalized power). **(B)** Probe localization based on stereotactic coordinates. **(C)** Refined probe positions after independent atlas-based alignment of each probe to maximize correspondence between clusters and anatomical regions.

Power feature extraction and clustering were performed jointly across the two probes to assess the consistency of the resulting spectral organization. The band-specific power maps shown in Figure 6A closely resemble those obtained with SiNAPS probes implanted in the hippocampus, despite differences in probe technology and electrode layout, indicating that LFP-LOC is robust to these factors. Output clusters were not perfectly consistent between the two probes, potentially reflecting minor device-to-device differences or local effects of the surgical implantation procedure. In particular, small differences in insertion angle or acute tissue damage at the two implant sites may differentially affect the recorded signals, resulting in subtle variations in the extracted spectral features. Nevertheless, a clear correspondence between clusters and anatomical features was observed.

To refine probe localization, atlas matching was performed independently for each probe, allowing greater flexibility in adjusting probe position. This procedure resulted in improved alignment between electrode clusters and brain anatomy (Figures 6B,C). Together, these results demonstrate that the proposed pipeline generalizes effectively to Neuropixels 2.0 recordings, despite their more limited spatial coverage compared with eight-shank SiNAPS probes.

## 4 Discussion

In this work, we introduce LFP-LOC, a simple, interpretable, and computationally light framework for anatomical localization of high-density neural probes based on the spatial distribution of LFP spectral power. By leveraging well-established relationships between low-frequency electrophysiological activity and brain structure, our approach enables rapid, data-driven validation of probe placement across diverse brain regions, probe technologies, electrode layouts, and experimental conditions.

A key strength of LFP-LOC lies in its generality. Unlike approaches that rely on region-specific electrophysiological signatures or supervised learning pipelines trained on annotated datasets, our framework operates without prior assumptions about local circuit organization and does not require training or fine-tuning. Across cortical and subcortical structures, including hippocampus, visual cortex, and medial prefrontal cortex, we consistently observed that electrodes clustered by LFP band power aligned with known anatomical boundaries. This robustness suggests that spatial variations in LFP spectral composition capture fundamental properties of brain organization.

The interpretability of LFP-LOC represents an important advantage over recent machine learning alternatives. By explicitly using power in canonical LFP frequency bands, the resulting features and clusters can be directly related to known physiological processes, such as laminar differences in synaptic input, network oscillations, and population firing dynamics. This transparency facilitates intuitive validation by experimenters and supports hypothesis-driven investigations of regional electrophysiological organization. A potential concern when using signal-derived features for anatomical localization is circularity between localization and subsequent signal interpretation. In LFP-LOC, clustering is fully unsupervised and anatomical labels are introduced only in a later atlas-matching step. Moreover, clusters do not necessarily correspond one-to-one with anatomical regions, and localization can be performed on short data segments independent from those used for downstream analyses, thereby mitigating potential circularity concerns. Because localization relies on user-selected LFP data, the risk of circular inference or functionally driven clustering cannot be fully excluded and should be considered when interpreting results.

Interestingly, our results also highlight the importance of high-density spatial sampling for accurate electrophysiological anatomy mapping. Spatial downsampling analyses demonstrated that performance degrades as electrode spacing increases, particularly in anatomically complex regions such as the hippocampus. Different and complementary approaches to LFP-LOC, for example examining functional responses rather than spontaneous activity, might extend the validity of this observation in different anatomical regions such as the visual and sensorimotor cortices. These findings further underscore the value of dense CMOS-based probes with wide spatial coverage.

Importantly, we show that reliable spectral power estimates can be obtained from relatively short recording durations. Across datasets, power features converged within approximately 10–20 s, enabling potential intraoperative validation and adjustment of implant location, either during acute recordings or in early phases of chronic implantations. The variability in the time required for stable power feature estimates may depend on the difference between target regions (e.g., hippocampus vs. visual cortex) and on experimental conditions (e.g., head-fixed vs. anaesthetized). Further analysis will be required to evaluate the individual impact of these parameters on feature estimates and to determine the minimum time required in different settings. In general, the opportunity of matching power features to the atlas using a relatively short (<30 s) recording duration is expected to substantially reduce experimental failure rates associated with stereotactic mistargeting and reliance on *post hoc* histological reconstruction. While the current implementation operates offline, the short data requirements and low computational cost allow the method to be applied within a few minutes during surgery, making it compatible with intraoperative workflows in practice. Integration of the pipeline into real-time acquisition systems represents a feasible and valuable extension that could provide immediate feedback during probe insertion and facilitate broader adoption, particularly in high-throughput experimental settings.

The integration of atlas-based refinement further enhances the practical utility of the approach. By systematically exploring local shifts around user-defined stereotactic coordinates and selecting the configuration that maximizes consistency between electrophysiological clusters and anatomical labels, LFP-LOC provides a principled means to validate and refine probe localization within a constrained neighborhood.

Several limitations should be acknowledged. First, the correspondence between electrophysiologically derived clusters and anatomical regions is not necessarily one-to-one. Although in most cases cluster borders appear to align well with known anatomical features, individual clusters may span multiple anatomically defined subregions or, conversely, a single anatomical region may be subdivided into multiple clusters. This can be partly explained by the fact that LFP spectral properties typically vary gradually rather than abruptly across anatomical boundaries. Second, the proposed approach assumes that the initial implant position is close to the intended stereotactic coordinates, so that most clusters are initially assigned to the correct anatomical region. Large targeting errors may therefore not be corrected by the local atlas-based refinement. Third, probes with limited lateral coverage, such as single-shank devices, provide reduced spatial context, resulting in a lower localization reliability compared with multi-shank probes. Finally, atlas-based validation remains inherently limited by inter-animal anatomical variability, as the use of a population-averaged template does not fully capture subject-specific differences in brain size and geometry. While in practice this variability appears sufficiently limited to enable reliable probe localization, particularly when multiple clusters across the probe provide redundant spatial constraints, it may still introduce inaccuracies in electrode-to-region assignment. Future work could address this limitation by incorporating subject-specific scaling (e.g., based on bregma-lambda distance) or by integrating deformable atlas registration approaches.

More broadly, the reliability of LFP-LOC depends on signal quality: although small localized artifacts are unlikely to substantially affect probe refinement because the atlas-matching step is dominated by larger and spatially consistent cluster-anatomy correspondences, widespread artifacts, including common-channel contamination that produces channel-dependent spectral distortions, may obscure anatomically relevant spatial variability and lead to inaccurate clustering and localization results. In this context, CAR may help attenuate shared-channel contamination and is therefore provided as an optional preprocessing step in the LFP-LOC package; however, its use is only suggested when such artifacts are suspected, since in the absence of evident contamination it tends to increase the number of clusters without substantially changing the final anatomical placement (Supplementary Figure 8; Supplementary Table 4).

A direct quantitative comparison between LFP-LOC-derived localization and histological reconstruction would provide an important benchmark for assessing accuracy and remains an important direction for future work. However, it is important to note that histological approaches, while widely regarded as a reference standard, have intrinsic limitations when used for precise electrode localization. These include tissue deformation during processing, uncertainty in slice alignment, and limited resolution in mapping individual recording sites to anatomical structures. As a result, quantitative comparisons at the scale of individual electrodes are not straightforward and may themselves be affected by non-negligible spatial errors. Future studies combining LFP-LOC with complementary validation strategies, including histology and imaging-based approaches, could provide a more comprehensive assessment of localization accuracy.

Overall, LFP-LOC fills an important methodological gap by providing a fast, interpretable, and technology-agnostic method to infer anatomical context directly from electrophysiological recordings. Beyond enabling reliable probe localization, it supports more consistent data annotation, cross-experiment comparison, and downstream analyses such as region-specific cell-type classification. More broadly, the approach offers a bridge between high-resolution mesoscale electrophysiology and macroscopic spectral mapping techniques commonly used in clinical and translational research, where spatial resolution is limited but spectral information is central.

## Data Availability

Requests to access these datasets should be directed to alberto.perna@iit.it.
