# Supplementary material for: LFP-LOC: an LFP power–based method for validating the anatomical placement of high-density neural probes in rodents
Source: Front Neurosci. 2026 May 11;20:1816533. doi: 10.3389/fnins.2026.1816533 (PMC13199280; doi:10.3389/fnins.2026.1816533)
Supplement: Supplementary file 1 [file Data_Sheet_1.docx]

Supplementary Material for LFP-LOC: an LFP power–based method for the anatomical localization of high-density neural probes

**
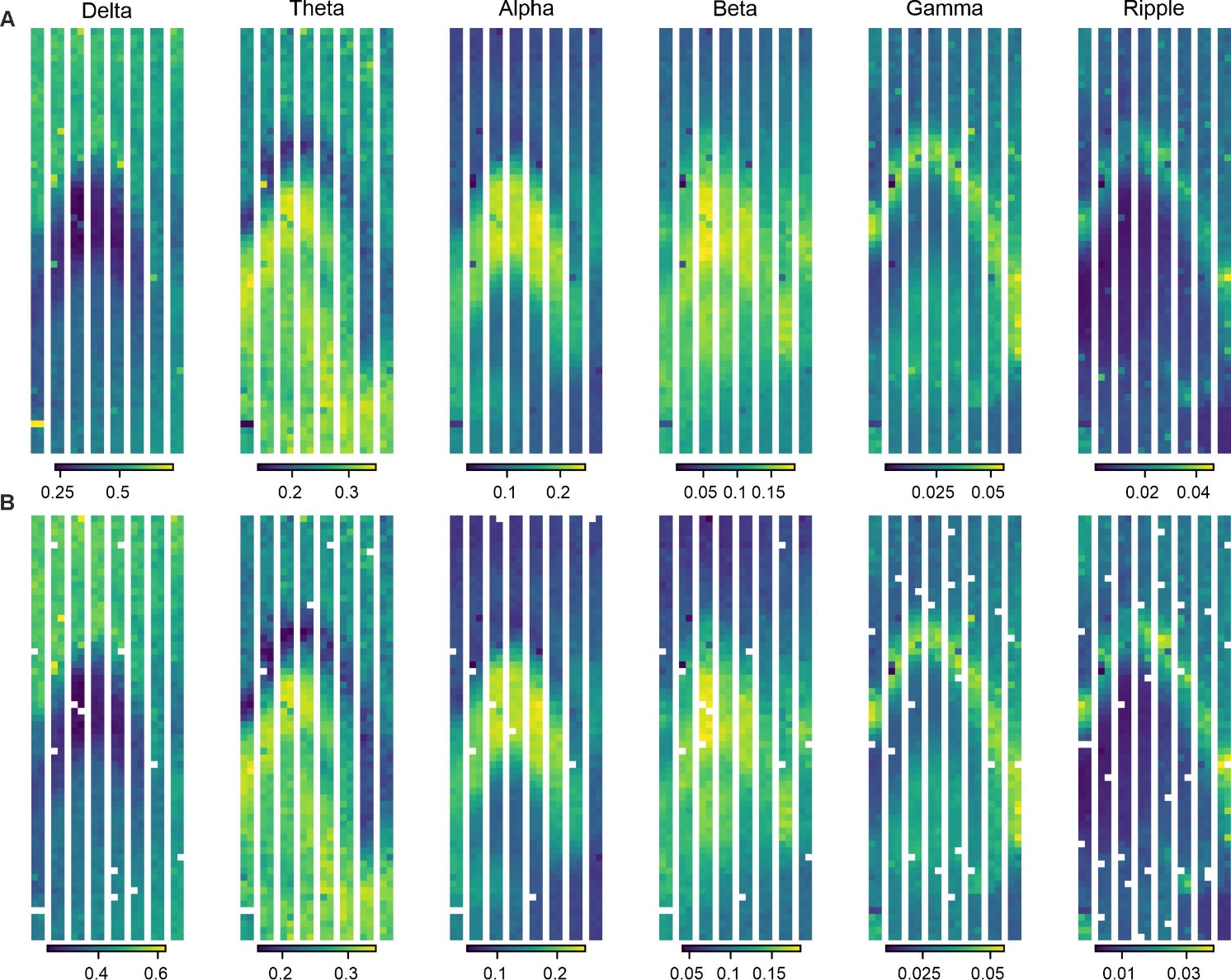
**

**Supplementary Figure 1 - Outlier detection in power features.** **(A)** Raw band-limited LFP power mapped across probe geometry (normalized power) in the eight-shank hippocampus recording. **(B)** Result of local outlier detection based on z-score thresholding.

**
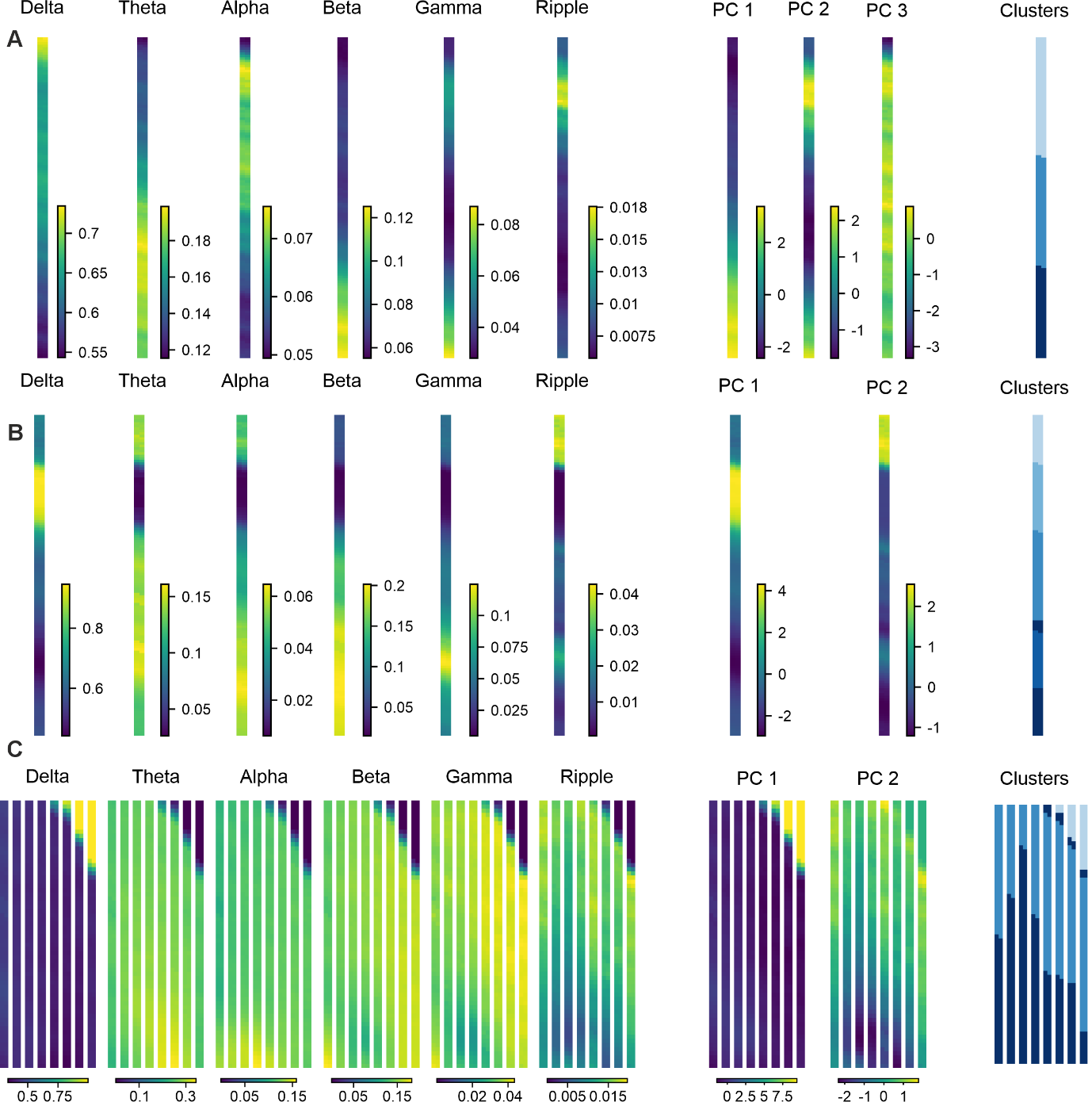
**

**Supplementary Figure 2 - Feature extraction and clustering across datasets.** Smoothed power features (normalized power), principal components (a.u.), and clustering results (cluster labels) for additional datasets: **(A)** Medial prefrontal cortex, single-shank probe, **(B)** Visual cortex, single-shank probe, and c, Visual cortex, eight-shank probe.


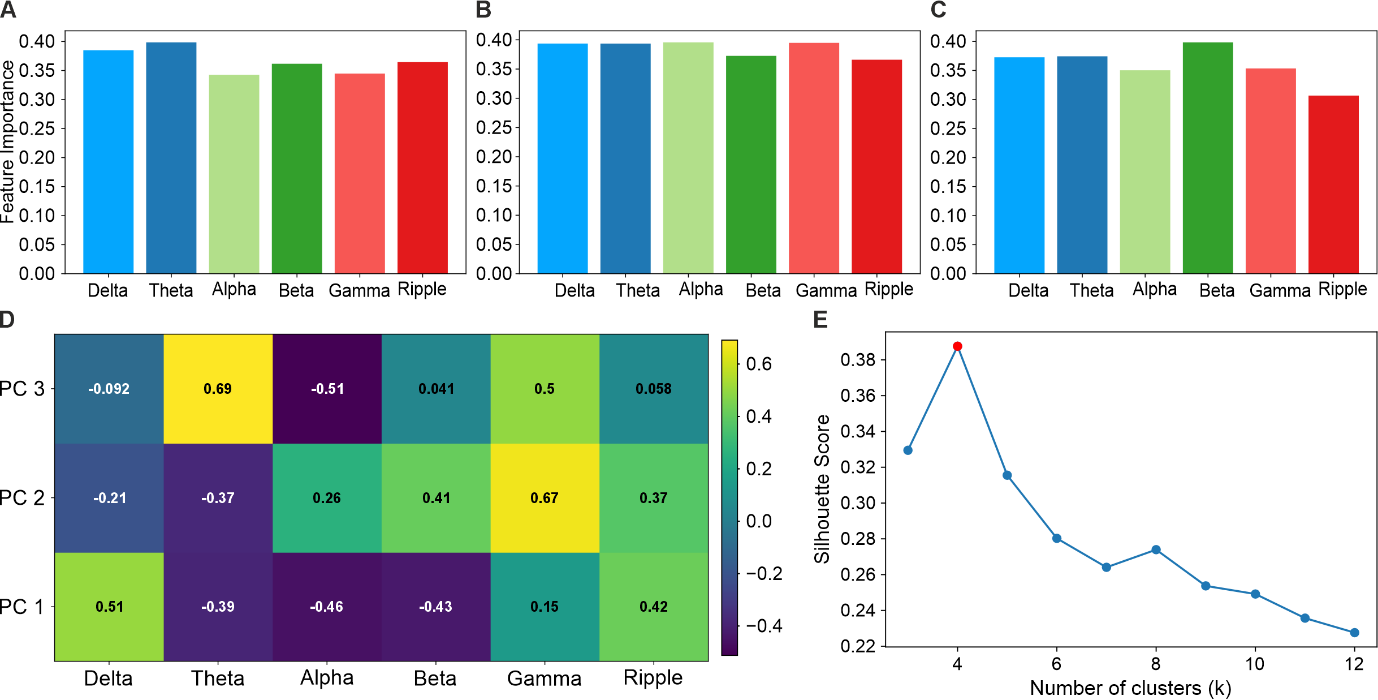


**Supplementary Figure 3 - Feature importance and optimal cluster count.** Relative contribution of spectral bands to PCA-reduced features for **(A)** Medial prefrontal cortex (single shank), **(B)** Visual cortex (eight shanks), and **(C)** Visual cortex (single shank). **(D)** PCA loading coefficients for band power features in the eight-shank hippocampus recording. **(E)** Selection of the optimal number of clusters based on silhouette score for the eight-shank hippocampus recording.

**
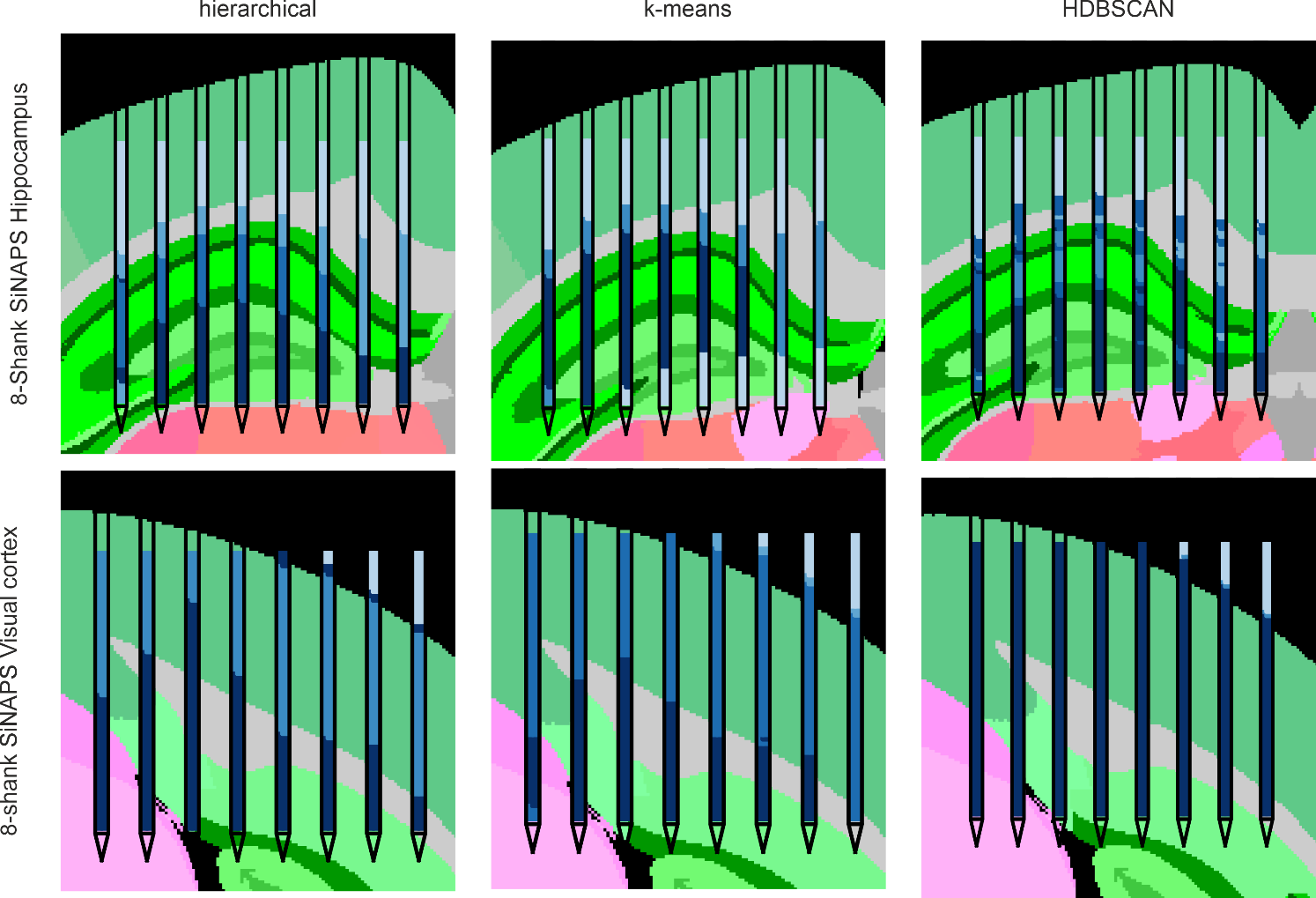
**

**Supplementary Figure 4 – Examples of clustering results.** Hierarchical, k-means and HDBSCAN clustering methods applied to the 8-Shank SiNAPS hippocampus (top) and visual cortex (bottom) recordings. Hierarchical and k-means provide more robust and anatomically consistent clusters compared to HDBSCAN


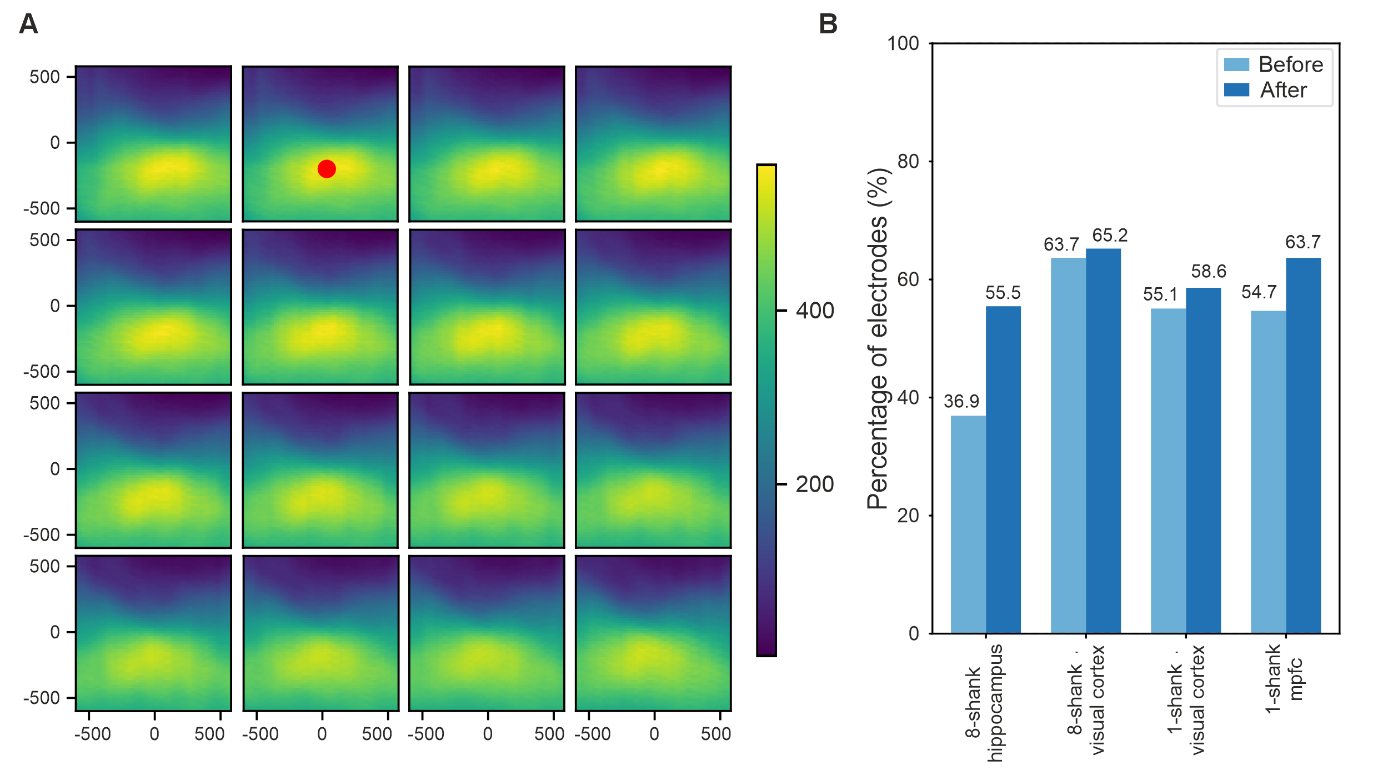
**Supplementary Figure 5 - Atlas-matching optimization matrices. (A)** Heatmaps showing the number of electrodes assigned to anatomically consistent regions as a function of probe shifts along mediolateral (x-axis) and dorsoventral axes (y-axis) for different coronal planes (matrices). The red marker indicates the shift combination yielding the best anatomical match. **(B)** percentage of electrodes in the appropriate region before and after probe adjustment in the different datasets.


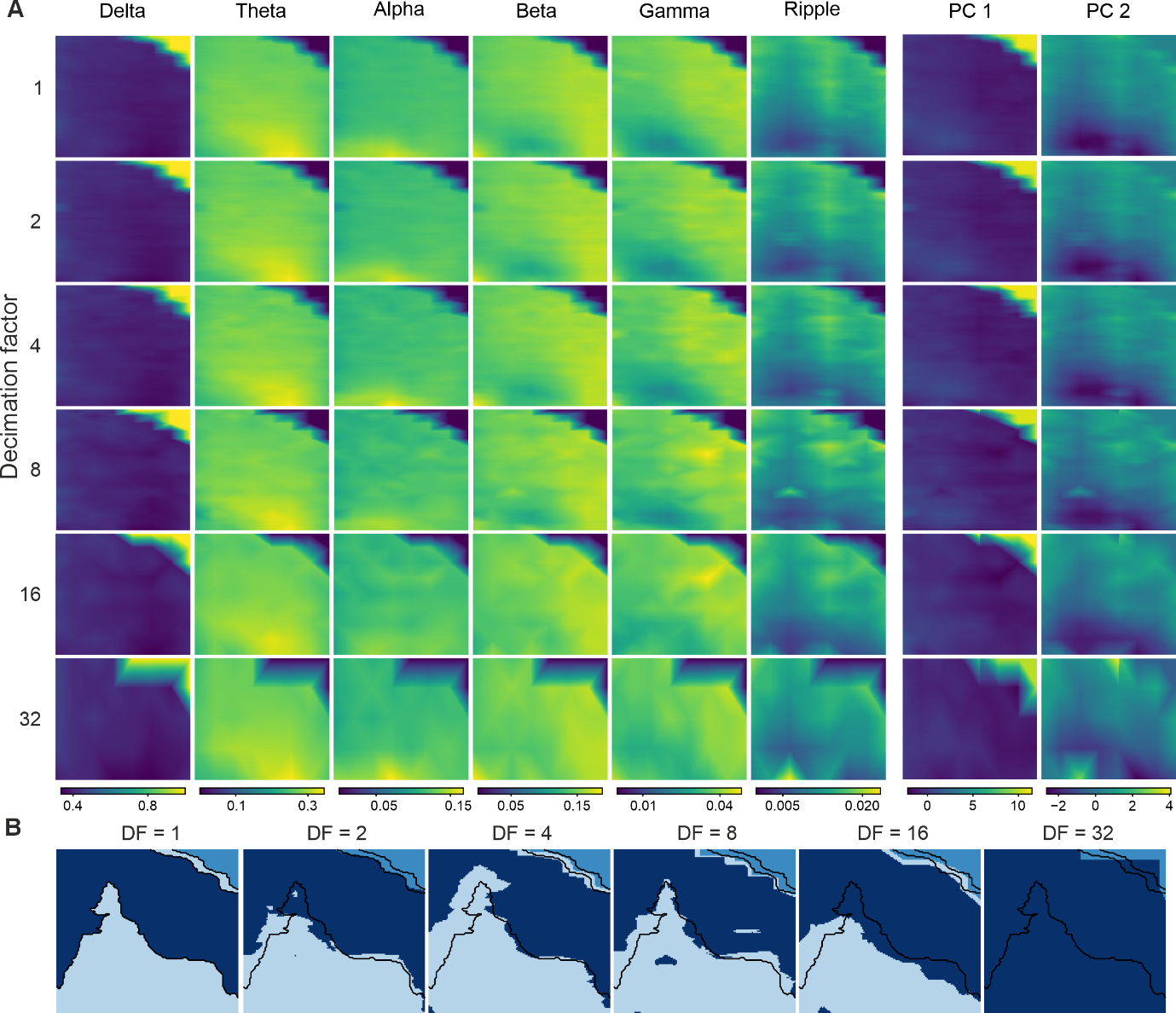


**Supplementary Figure 6 - Spatial downsampling in eight-shank visual cortex recordings. (A)** Band-limited power maps (normalized power) and principal components (arbitrary units) as a function of spatial decimation factor. **(B)** Corresponding cluster assignments; black outlines indicate full-resolution clusters (DF = 1).


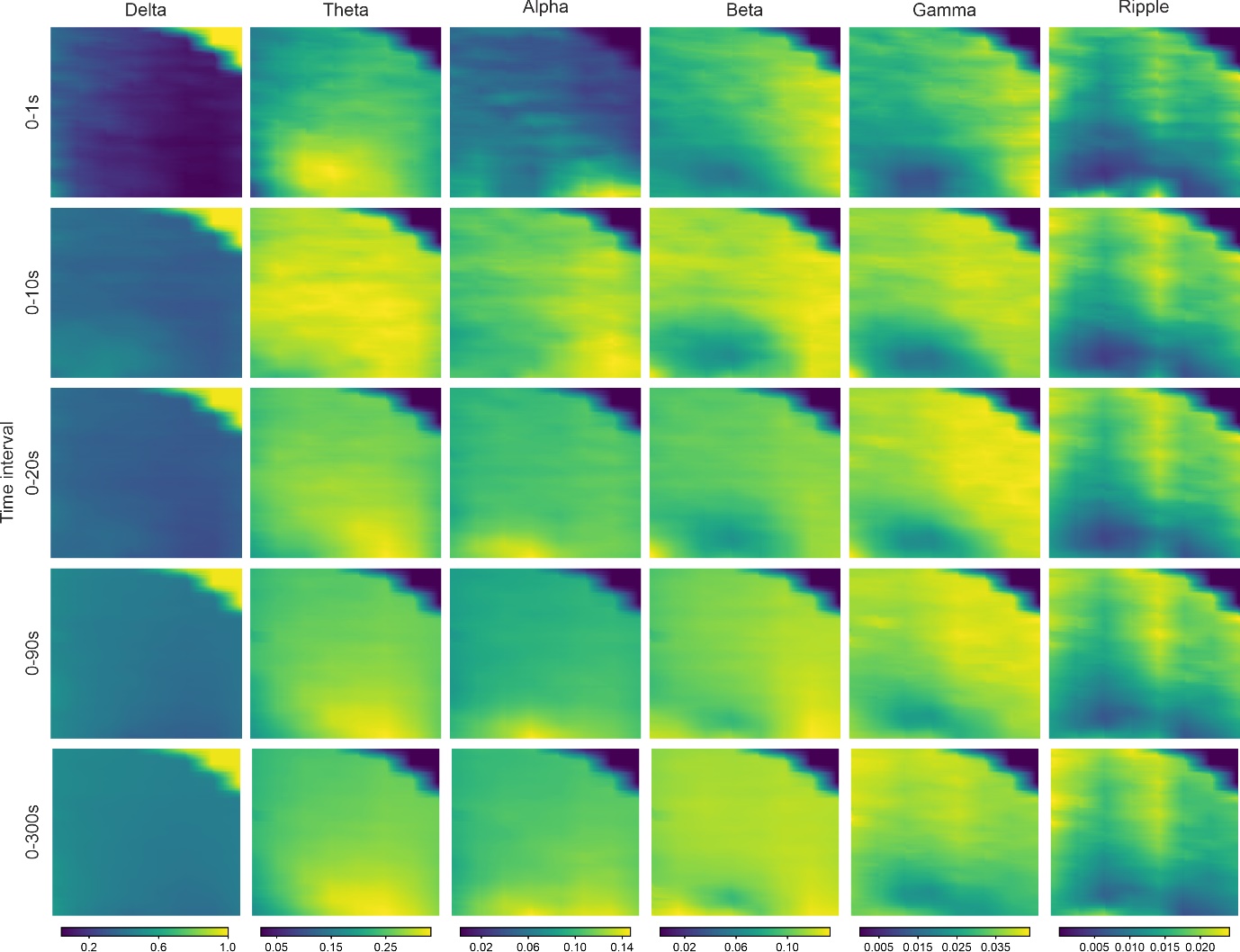


**Supplementary Figure 7 - Temporal evolution of spectral power features.** Example of band-specific power estimates (normalized power) over time for an eight-shank visual cortex recording, illustrating the stability of features used for PSD estimation after 10-20 seconds.


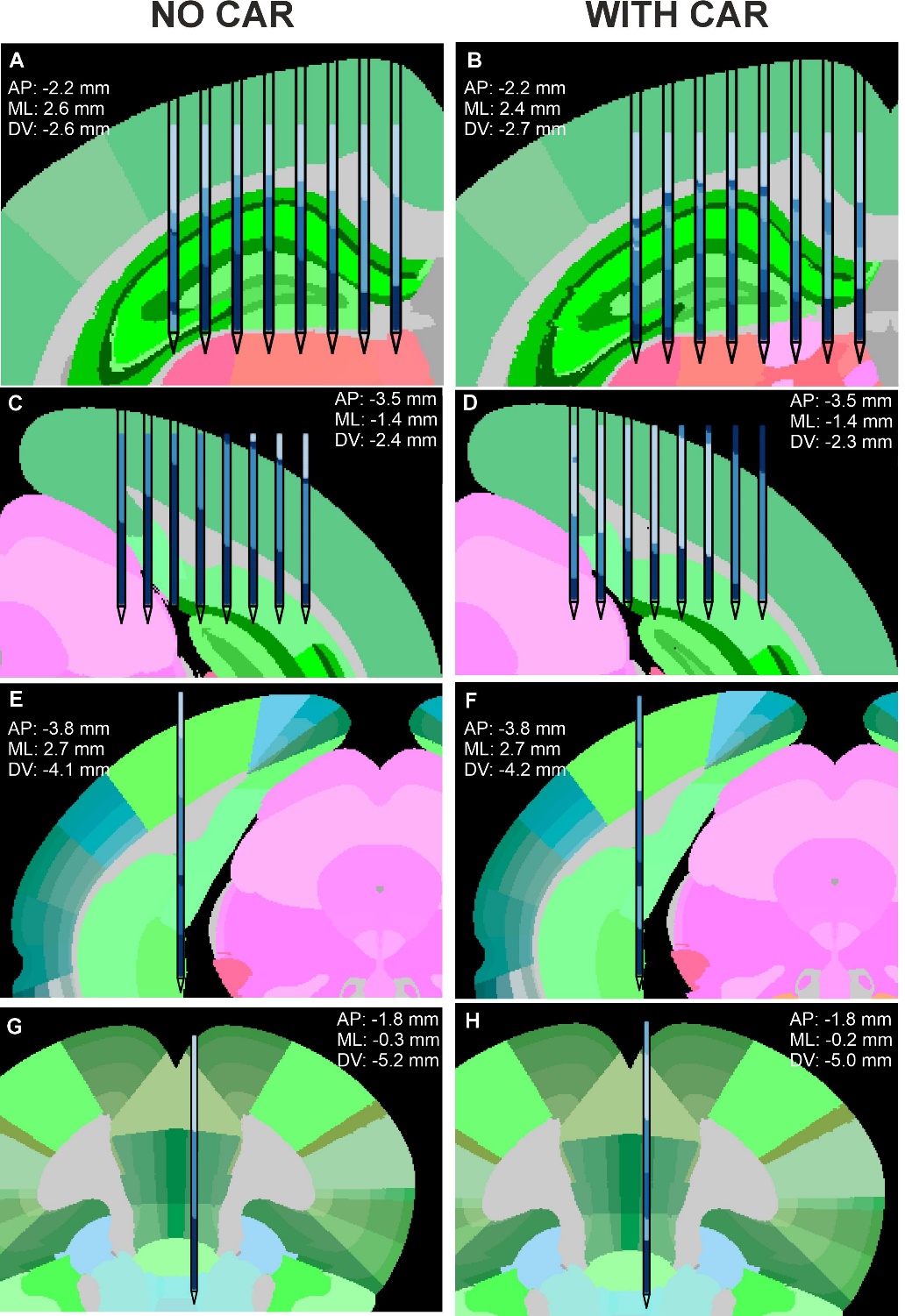


**Supplementary Figure 8 - Effect of common average referencing (CAR) on clustering-based atlas matching and probe refinement.** Comparison of atlas-based probe placement obtained without CAR (left column) and with CAR (right column) across all SiNAPS datasets analyzed in this study. Panels A–B: hippocampus, eight-shank probe; C–D: visual cortex, eight-shank probe; E–F: visual cortex, single-shank probe; G–H: medial prefrontal cortex, single-shank probe. In each panel, the probe is overlaid on the reference atlas at the refined AP, ML, and DV coordinates, and electrode clusters are shown along the shank(s). Across datasets, applying CAR in the absence of evident artifacts generally increased the number of clusters identified. However, the spatial borders of the clusters and their correspondence with the underlying anatomy remained broadly similar, and the final refined probe placement was only minimally affected. Quantitative differences in probe position with and without CAR are summarized in Supplementary Table 4.

| **Localization algorithm** | **Electrode type** | **Electrode size** | **Intra-operative viability** | **Required signal duration** | **Brain anatomy** | **Generality** |
| --- | --- | --- | --- | --- | --- | --- |
| **LFP-LOC**  **(This work)** | Tissue-penetrating MEAs | Micro (<14 µm site width; high-density probe electrodes) | Yes | 20–30 s | Template | Local |
| **GridLoc (Branco et al. 2018)** | Surface ECoG grids | Macro (1–1.3 mm exposed diameter) | Yes | 90–180 s | Native (pre-op MRI ± MRA / gadolinium MRI; validated against CT/photos) | Local |
| **Lead-OR**  **(Oxenford et al. 2022)** | Depth electrodes / DBS trajectories | Microelectrodes + macroelectrodes | Yes | ≥4 s artifact-free microelectrode recording segments | Native + template atlases warped to patient space | Local |
| **Miyagi et al., 2009** | Depth DBS electrode | Macro (1.27 mm diameter contacts, 1.5 mm length) | Yes | 10 s per depth step | Native (patient MRI/CT with intra-op refinement) | Local |

Supplementary Table 1 - Comparison of LFP-LOC with other localization algorithms for different electrode technologies

| Brain Region | Condition | Probe Type | 1st Percentile (µV²/Hz) | 99th Percentile (µV²/Hz) | 1st Percentile (dB) | 99th Percentile (dB) |
| --- | --- | --- | --- | --- | --- | --- |
| Hippocampus | Head-fixed | SiNAPS (8-shank) | 3.09 × 10² | 2.71 × 10³ | 24.9 | 34.3 |
| mPFC | Freely moving | SiNAPS (single-shank) | 7.15 × 10³ | 1.99 × 10⁴ | 38.5 | 43.0 |
| Visual cortex | Anesthetized | SiNAPS (8-shank) | 3.36 | 5.54 × 10⁴ | 5.26 | 47.1 |
| Visual cortex | Anesthetized | SiNAPS (single-shank) | 2.17 × 10⁻⁹ | 3.05 × 10³ | −86.6 | 34.8 |

**Supplementary Table 2 -** Distribution of LFP power spectral density values across datasets. For each dataset, the 1^st^ and 99^th^ percentiles of PSD values in the 1–300 Hz range are reported in both linear units (µV²/Hz) and decibels (dB). Percentiles are used to provide a robust estimate of the dynamic range while minimizing the influence of outliers.

| Brain Region | Condition | Excluded Electrodes (n) | Total Electrodes  (n_el * n_features) | Excluded (%) |
| --- | --- | --- | --- | --- |
| Hippocampus | Head-fixed | 94 | 6144 | 1.53% |
| mPFC | Freely moving | 32 | 1536 | 2.08% |
| Visual cortex | Anesthetized | 44 | 1536 | 2.86% |
| Visual cortex | Anesthetized | 101 | 6144 | 1.64% |

**Supplementary Table 3 -** Summary of electrodes excluded during local outlier detection. Values are reported for each dataset, including the total number and percentage of excluded electrodes, as well as the mean number of excluded electrodes per 20-channel window.

|  | No CAR | | | With CAR | | |  |
| --- | --- | --- | --- | --- | --- | --- | --- |
| Dataset | AP | ML | DV | AP | ML | DV | Distance |
| Eight-shank Hippocampus | -2.16 | 2.56 | -2.65 | -2.22 | 2.4 | -2.71 | 0.18 |
| Eight-shank Visual cortex | -3.5 | -1.38 | -2.4 | -3.48 | -1.4 | -2.34 | 0.066 |
| Single-shank Visual cortex | -3.8 | 2.72 | -4.08 | -3.8 | 2.72 | -4.18 | 0.10 |
| Single-shank mPFC | 1.8 | -0.26 | -5.2 | 1.8 | -0.22 | -5.02 | 0.18 |

**Supplementary Table 4.** Comparison table of the refined insertion coordinates for the different datasets with and without the application of CAR
